# Choroidal melanoma tumor profile and treatment pattern for newly diagnosed patients at a reference public hospital in Sao Paulo, Brazil

**DOI:** 10.1186/s12886-022-02742-y

**Published:** 2022-12-28

**Authors:** Arthur Gustavo Fernandes, Jorge Henrique Cavalcante Tavares, Ana Marisa Castello Branco, Melina Correa Morales, Rubens Belfort Neto

**Affiliations:** 1 Departament of Ophthalmology and Visual Sciences, Escola Paulista de Medicina, Universidade Federal de São Paulo – UNIFESP, Rua Botucatu, 816, 04023-062, SP Sao Paulo, Brazil; 2 Department of Anthropology and Archaeology, University of Calgary, Calgary, AB Canada

**Keywords:** Delivery of health care, Choroidal neoplasms, Eye neoplasms, Ophthalmology, Public health

## Abstract

**Background:**

Choroid, ciliary body, and iris melanomas are often grouped as uveal melanoma, the most common intraocular primary malignancy. The purpose of the current study was to analyze the tumor profile of newly diagnosed cases of choroidal melanoma at a reference center in Sao Paulo, Brazil, and to investigate the frequency of eyes treated by enucleation that could have been treated with brachytherapy if available in the service.

**Methods:**

Medical records of patients referred to our service with initial diagnostic hypothesis of choroidal melanoma from July 2014 to June 2020 were analysed on demographics, diagnosis confirmation, tumor measurement by ultrasonography and established treatment. Data were evaluated on clinical and demographic characteristics as age, sex, affected eye, ultrasound parameters, and treatment management of patients with clinically diagnosed choroidal melanoma. Among the patients submitted to enucleation, we investigated how many could have been selected to receive brachytherapy.

**Results:**

From the 102 patients referred with the choroidal melanoma diagnosis hypothesis, 70 (68.62%) were confirmed. Mean measurements from the tumors in millimetres were: 9.19 ± 3.69 at height and 12.97 ± 3.09 by 13.30 ± 3.30 at basal. A total of 48 cases (68.57%) were enucleated, 8 (11.43%) were treated by brachytherapy in a different service, and 14 patients (20.00%) returned for enucleation at their original referral center. Out of the 48 patients enucleated, 26 (54.17%) could have been selected to brachytherapy treatment.

**Conclusions:**

The results indicate a late diagnosis of choroidal melanoma cases referred to our service. Most enucleated cases could have been treated with brachytherapy if it was broadly available at the national public health insurance. Further public health political efforts should focus on early diagnosis and better quality of life post-treatment for oncologic patients.

## Background

Choroid, ciliary body, and iris melanomas are often grouped as uveal melanoma, the most common intraocular primary malignancy [1–3]. The age-adjusted incidence of uveal melanoma in the North America is 4.3 per million, which has remained relatively stable over the last 5 decades [4–6]. Mean age of onset is reported at 60 years and known risk factors includes fair skin, light-colored eyes, advanced age, preexisting choroidal nevus, congenital ocular melanocytosis and BAP1 gene mutation [2, 3, 7–9]. Unlike cutaneous melanoma, ultraviolet light exposure has no confirmed association to increasing risk of developing choroidal melanoma [2].

Choroidal melanoma alone represents 85% of the uveal melanoma cases [1, 10]. The diagnosis is usually based in clinical and ultrasound characteristics. The most frequent symptoms are low visual acuity, photopsies, floaters and scotomas, usually reported late and only noticed when there is significant growth or complication of the neoplasm, such as perilesional retinal detachment [3, 7, 11]. The typical tumor appears as a solid, single, unilateral, pigmented, elevated lesion, with orange pigment, in the shape of a dome (75%), mushroom (20%) or difuse (5%), with its own vascularization, and may appear with associated retinal detachment or, rarely, with vitreous hemorrhage [7, 11]. Ultrasonography helps in the diagnosis showing an intraocular mass of low to medium reflectivity, homogeneous, with signs of internal vascularization, forming acoustic hollowness, kappa angle, and choroidal excavation [12, 13].

Historically, the mainstay of treatment for primary melanoma has been enucleation, an approach that essentially consists of the globe removal [1, 2]. Orbital implants placement often leads to excellent results in terms of cosmesis, but the patients go completely blind as the eye is removed [2]. In the last decades, however, radiotherapy has acquired an increasingly important role to the point of becoming the first-line modality to treat small and medium sized melanomas [14]. Chemotherapy is generally not an option as it is not efficient against this type of tumor [15, 16].

Brachytherapy is a treatment modality for choroidal melanomas in which a radioactive implant is sutured to the sclera. This structure consists of radioactive seeds of a specific isotope attached to a protective radiopaque plaque. As a consequence, radiation can be directed almost exclusively to the desired area, minimizing adverse effects to adjacent tissues [17]. Modern plaques typically utilize either low-energy photon-emitting seeds (125I, 131Cs) assembled inside a gold alloy or stainless steel backing or beta particle-emitting sources (106Ru, 90Sr) attached to silver plaques, or both types of energy (198Au) [18, 19]. Dosimetry, plaque choice, as well as number of plaque attached days’ calculation, are made previously to the surgery, according to type and half-life of the radionuclide and to tumor measurements. Apical tumor height is considered the most important value to calculate radiation dose because, depending on the radionuclide, there are limits for tissue thickness that can be effectively penetrated by the emitted energy. Ruthenium (106Ru) plaques, for example, can only be used for tumors with less than 5 mm in height, while iodine (125I) plaques are safely used for bigger melanomas [17, 20, 21]. In Brazil, however, brachytherapy is hardly available to the public health care system, and most ocular oncology public centers tend to perform enucleation as primary treatment for uveal melanomas.

The present study aimed to investigate the tumor profile of patients diagnosed with choroidal melanoma at the Ocular Oncology division from the Department of Ophthalmology UNIFESP and to investigate the frequency of eyes treated by enucleation that could have been treated with brachytherapy, if it was available in the service.

## Methods

A retrospective study was carried out by medical chart review of the patients referred to the ocular oncology division from the Federal University of São Paulo (UNIFESP) with an initial diagnostic hypothesis of choroidal melanoma, during the period of July 2014 to June 2020. This study was approved by the UNIFESP Institutional Review Boards (#4.355.635) and was carried out in accordance with the tenets of the Declaration of Helsinki.

Patients referred to UNIFESP went through a detailed eye examination including slit-lamp anterior segment biomicroscopy, fundoscopy and ultrasonography to confirm the choroidal melanoma diagnosis.

Individuals with confirmed diagnosis were advised about treatment options for choroidal melanoma but were informed that the only curative treatment available in our service was enucleation surgery. It was given the option to the patient to look for other treatment modality outside the public health care system if wanted. Tumors were classified based on the American Joint Committee on Cancer (AJCC) guidelines [22]. Brachytherapy was considered an option management when tumor height was less than 10 mm considering a limit of 5 mm for Ruthenium plaque and limit of 10 mm for Iodine plaque. The brachytherapy indication also considered extraocular extension, visual potential, pain status, and other ocular comorbidities, as recommended by the American Brachytherapy Society guidelines [23].

Statistical analyzes were performed using Stata/SE Statistical Software, Release 14.0, 2015 (Stata Corp, College Station, Texas, USA). Data was analyzed on clinical and demographic characteristics as age, sex, affected eye, ultrasound parameters, and treatment management of patients with clinically diagnosed choroidal melanoma. Ultrasound measurements included tumor height and basal diameters (antero-posterior and latero-lateral) in millimeters (mm). The outcome was determined as the percentage of eyes that could have been treated with brachytherapy but were treated with enucleation.

## Results

Over the study period, a total of 102 patients were referred to the ocular oncology division with an initial hypothesis of choroidal melanoma, of which 70 (68.62%) had diagnostic confirmation. Out of the 32 cases of pseudomelanomas, most cases were due to choroidal nevus (40.62%), subretinal hemorrhages (18.75%), and subretinal neovascular membrane (18.75%). Table 1 shows the characteristics of the 70 patients with confirmed diagnosis of choroidal melanoma included in the analysis.

**Table 1:** Sample descriptive analysis

Characteristics	
**Sex**; *N(%)*	
Male	37 (52.86)
Female	33 (47.14)
**Origin**; *N(%)*	
São Paulo State	60 (85.71)
Other region	10 (14.29)
**Age in years**; *mean ± std*	61.67 ± 13.37 (62.00)
**Affected eye**; *N(%)*	
Right eye	37 (52.86)
Left eye	33 (47.14)

**Fig. 1:**
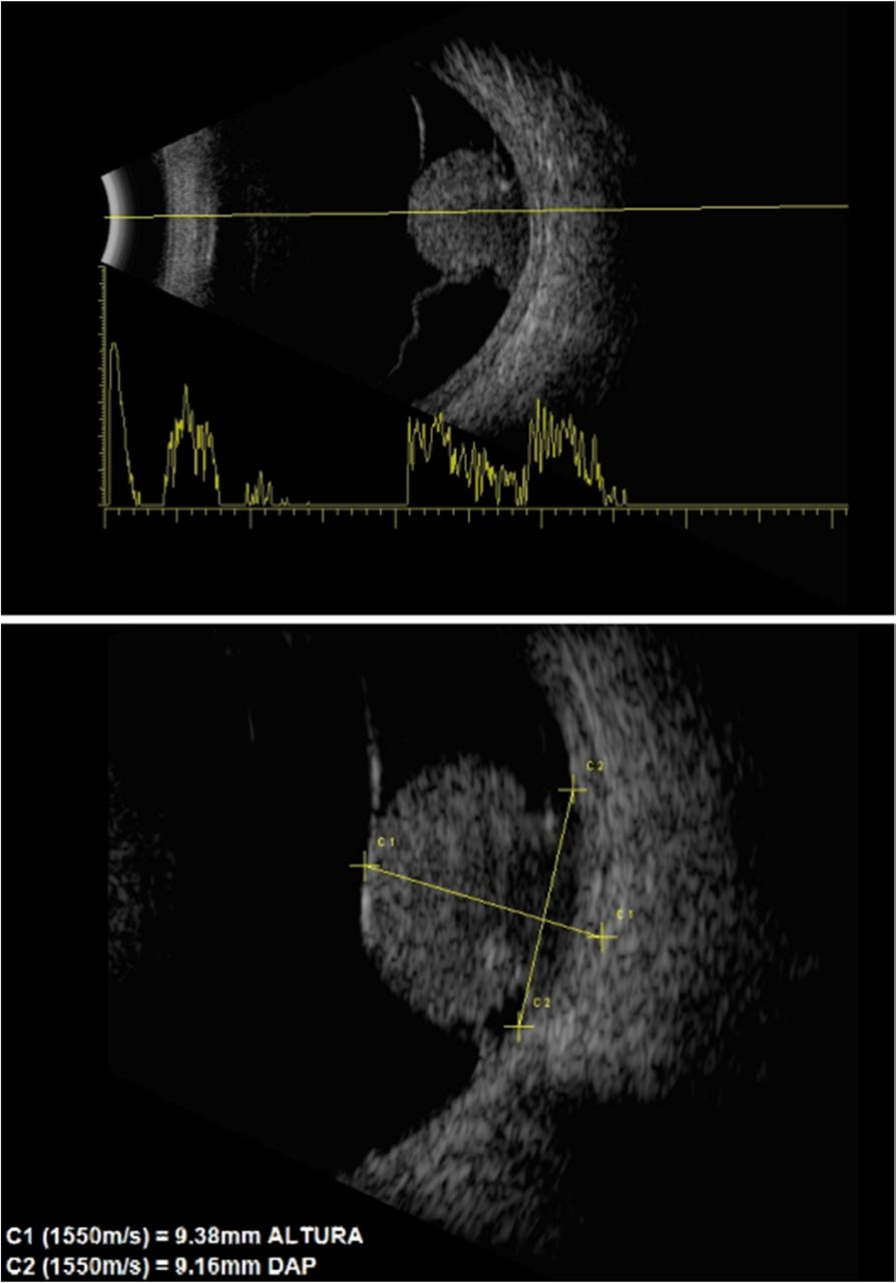
Ocular ultrasound from a 16 years old female patient with a mushroom shaped choroidal melanoma. Superior image shows a transversal B-scan on posterior pole – the lesion has medium to low reflectivity decreasing towards the tumor base, indicating kappa angle sign. Inferior image shows a longitudinal B-scan – the lesion presents acoustic hollowness at its base, tumor dimensions were 9.38 mm of height and 9.16 mm of circumferential basal diameter

Out of the 70 patients, 2 (2.80%) presented hepatic metastasis at the ocular diagnosis. The mean tumor height was 9.19 ± 3.69 (median: 9.00) millimeters and the mean basal diameter was 12.97 ± 3.09 (12.99) millimeters for antero-posterior and 13.30 ± 3.30 (12.80) millimeters for latero-lateral. Figure 1 shows an ultrasound of a patient with choroidal melanoma confirmation.

Among the confirmed diagnosis patients, 8 (11.43%) underwent brachytherapy in another service and 14 (20.00%) returned for enucleation at the referral center. Therefore, 48 (68.57%) remained at our service and all of them were submitted to enucleation of the eye globe affected by melanoma with placement of a primary orbital implant. Table 2 shows the characteristics of these eyes.

**Table 2:** Characteristics of enucleated eyes

	N (%)
**Tumor apical height**	
Small (<=5 mm)	1 (2.08)
Medium (5.1 – 10 mm)	25 (52.09)
Large (> 10 mm)	22 (45.83)
**Largest basal diameter**	
> 20 mm	16 (33.33)
< =20 mm	32 (66.66)
**Sclera invasion by pathology**	
Yes	37 (77.08)
No	11 (22.92)
**Extraocular extension by pathology**	
Yes	1 (2.08)
No	47 (97.92)
**AJCC Classification**	
T1	1 (2.08)
T2	10 (20.83)
T3	30 (62.50)
T4	7 (14.58)

All cases were confirmed as melanoma by histopathology and were classified as epithelioid (60.42%), spindle (31.25%), and mixed (8.33%). Cases with extraocular extension confirmed by pathology were referred for radiotherapy adjuvant therapy.

Out of the total eyes submitted to enucleation, 26 (54.17%) had apical height less than 10 mm and therefore could be treated with brachytherapy using iodine-125 plaque, if it were available in our service. Still, 1 case (2.08%) showed an apical height of 4.9 mm and could be treated with brachytherapy using Ruthenium plaque.

## Discussion

The ocular oncology division at UNIFESP is one of the main centers specialized in ocular oncology treatment in Brazil through the national health insurance system SUS [24]. About 30% of patients referred to our service with an initial diagnostic hypothesis of choroidal melanoma were confirmed as not having the disease which reflects the challenges faced by general ophthalmologists due to the rarity of melanoma. Pseudomelanomas are a group of diseases that clinically simulate a choroidal melanoma leading to diagnostic ambiguity. According to the literature, the most frequent pseudomelanomas are choroidal nevus, peripheral exudative hemorrhagic chorioretinopathy, congenital hypertrophy of retinal pigment epithelium, idiopathic hemorrhagic detachment retina or pigment epithelium, circumscribed choroidal hemangioma, and age-related macular degeneration [7]. Most of the pseudomelanoma cases in the current study were compatible with choroidal nevus and subretinal hemorrhages secondary to choroidal neovascularization associated to age-related macular degeneration or polypoidal choroidal vasculopathy, which are described as important differential diagnosis.

The population demographics are in accordance with the international literature on choroidal melanoma. In terms of tumor profile, however, we observe a few discrepancies. Studies from North America and Finland have shown mean tumor heights of 5.5 and 6.0 mm, respectively, importantly lower than our results of 9.2 mm [11, 25]. Similarly, the larger basal diameters (LBD) of those studies were 11.1 and 11.3 mm while we observed a mean of 14.1 mm [11, 25]. These results indicate that patient with choroidal melanoma in Brazil take more time to get specialized care with an ocular oncologist which may reflect the low access of the population to eye health care. Similarly, a review of cases in Mexico found mean height of 10.9 mm and LBD of 13.5 mm also explained by the lack of access of the population to specialized eye care centers, a pattern that might be repeated in other countries from Latina America, reinforcing the health inequalities across the globe [26].

Besides the visual impairment that might be associated with the late diagnosis, patients with choroidal melanoma are at risk for metastatic disease to the liver, lung, and skin [7, 27]. Previous studies indicate that each millimeter of increase on tumor height is associated with an increased relative risk of metastasis of 2.22 (95%CI: 1.22–4.05) reinforcing the importance of early diagnosis [28]. The frequency of metastasis from choroidal melanoma is reported as 8, 15, and 25% at 3, 5, and 10 years from diagnosis, respectively, and therefore systemic monitoring including physical examination and liver function tests each 6 months and annual chest radiograph and liver imaging using MRI or ultrasonography are strongly recommended even after the ocular treatment [7]. Five-year melanoma related mortality is estimated in 16, 32 and 53% for small, medium, and large tumors [29]. While only about 3% of our cases presented systemic metastasis at the moment of the choroidal melanoma diagnosis, the risk of developing it on the following years is high due to tumors’ size. Patients treated in our service are followed up for metastasis risk performing liver imaging evaluation each 6 months.

Trials from the Collaborative Ocular Melanoma Study (COMS) have shown no statistically significant differences in death-related or melanoma metastasis between enucleation and brachytherapy treatments in patients with medium size tumors [30]. In terms of vision following treatment, however, there are substantial differences according to the chosen treatment. Enucleation leads to blindness right after the treatment once the eye is removed. Plaque brachytherapy has the advantage of offering chances of preserving visual functionality after treatment, even though radiation side effects may lead to vision loss in most patients. Cataracts, radiation maculopathy, radiation neuropathy or secondary complications similar to those of diabetic retinopathy with vitreoretinal and iris neovascularization are complications associated with brachytherapy [17]. Their severity depends on tumor characteristics such as proximity to the fovea or optic disc, apical height, basal diameter and consequently on the calculated radiation dose necessary to achieve tumor growth control [14, 17]. A case series involving 243 ocular melanomas treated with brachytherapy found that about three quarters of the patients ended up with legally blind after 5 years of follow-up [31]. New adjuvant treatments, however, seem to be able to lower the risk of visual impairment [17]. It’s worth mentioning, yet, that vision loss is usually gradual, meaning that this kind of treatment may allow months to years of useful visual acuity even for these patients with worse long-term visual outcomes. When comparing the different modalities of brachytherapy, the literature indicates better long-term visual prognosis and lower rates of radiation retinopathy for those eyes associated with Ruthenium therapy when compared to Iodine [21].

All choroidal melanomas treated in our service during the study period were conducted to enucleation and part of them could have been treated by brachytherapy if available. The Brazilian Unified Health System (Sistema Único de Saúde - SUS) was created in 1990 based on the following underlying principles: universal access to health services across all levels of care; equality of care, without prejudice and privilege of any kind; comprehensiveness; public participation; and political and administrative decentralization [32]. In that sense, treatment for choroidal melanoma has been offered without any cost to the patient aiming the disease cure. The fact that another treatment modality could benefit 60% of patients on preserving their eyes and perhaps their sight may open a discussion on the needs of more investment in technology, facilities and specialized human resource versus the priorities within the system. The cost of the brachytherapy treatment is estimated as 2.2 times the cost of the enucleation option [33]. The advanced cases treated in the service opens another discussion regarding access to eye care services through the national health system. The ocular oncologist figure is the last one on a chain of professionals that the patients have to visit on their way and the time between each referral also contributes to the late diagnosis. Along the last 30 years since creation, SUS has improved the access to healthcare mainly related to the increase supply for health services and human resources [34, 35], however, programs of specialized training as ocular oncology fellowships and knowledge disseminations strategies as seminars and punctual courses are needed to be stimulated so that no choroidal melanoma cases are left behind with misdiagnosis and life-threatened disease progression.

While the current study brings valuable information regarding tumor profile and treatment practices in Brazil, some limitations might be noted. The retrospective design is subject to the lack of specific information due to inconsistences on the data collection, so that statistics such as visual acuity and ocular comorbidities are not presented in the current report. The comparisons of outcomes after treatment between patients treated with enucleation and brachytherapy was not evaluated in this study as patients referred for other centers were not followed up in our service. Future multicenter longitudinal studies are encouraged in order to evaluate metastasis and death rates according to tumor profile and/or treatment regimen.

In conclusion, the tumor profile from patients treated in our service indicates a late diagnosis of choroidal melanoma. Most patients treated with enucleation could have been benefited from the treatment with brachytherapy that provide eye preservation and possibility of useful sight after treatment. Further public health political efforts should be made to offer an early diagnosis and better quality of life post-treatment for cancer affected patients.

## Data Availability

The data that support the findings of this study are available from the Ocular Oncology Division at UNIFESP but restrictions apply to the availability of these data, which were used under license for the current study, and so are not publicly available. Data are however available from the corresponding author upon reasonable request and with permission of the Ocular Oncology Division at UNIFESP.
